# Association of tumor-informed circulating tumor DNA dynamics with radiologic disease assessment in a single-center real-world sarcoma cohort

**DOI:** 10.1186/s12885-026-17180-y

**Published:** 2026-10-27

**Authors:** Branko Calukovic, Katrin Benzler, Martin Schulze, Florian Battke, Lars Zender, Ulrich M. Lauer, Saskia Biskup, Christoph K. W. Deinzer

**Affiliations:** 1 Department of Internal Medicine VIII - Medical Oncology and Pneumology, University Hospital Tübingen, Tübingen, Germany; 2 Center for Soft Tissue Sarcomas, GIST and Bone Tumors (ZWS) of the University Hospital Tübingen and the Comprehensive Cancer Center (CCC) Tübingen-Stuttgart, Tübingen, Germany; 3 DFG Cluster of Excellence 2180 ‘Image-Guided and Functional Instructed Tumor Therapy’ (iFIT), University of Tübingen, Tübingen, Germany; 4 Center for Human Genetics Tübingen, Tübingen, Germany; 5 CeGaT GmbH, Tübingen, Germany

**Keywords:** Soft tissue sarcoma, Bone sarcoma, Circulating tumor DNA (ctDNA), Liquid biopsy, Disease monitoring, Molecular residual disease, Real-world cohort

## Abstract

**Background:**

Sarcomas are rare and heterogeneous malignant tumors for which reliable blood-based biomarkers for longitudinal disease monitoring remain limited. Circulating tumor DNA (ctDNA) has emerged as a minimally invasive biomarker in several solid tumors, but its clinical utility in sarcoma remains incompletely defined because of biological heterogeneity, variable ctDNA shedding, and the limited availability of recurrent, panel-coverable genomic alterations across different subtypes.

**Methods:**

We conducted a retrospective real-world study of patients with bone and soft tissue sarcomas treated at a certified high-volume sarcoma center. Patients underwent tumor-normal molecular profiling to identify tumor-informed ctDNA targets. Longitudinal plasma monitoring was performed using a unique molecular identifier (UMI)-based targeted sequencing approach in patients with an identifiable target. ctDNA findings were compared with radiologic assessment, treatment response, and clinical outcomes. In two selected patients with extended longitudinal follow-up, whole-genome-based molecular residual disease (MRD) analysis was performed as an exploratory analysis.

**Results:**

Twenty patients were screened, of whom 14 (70%) had an identifiable tumor-informed ctDNA target and were included in the longitudinal analysis. A total of 67 longitudinal plasma samples were analyzed, with a median of 4 samples per patient (range: 2-10). A total of 67 paired liquid biopsy-radiologic assessments were included in the concordance analysis. Concordant assessments were observed in 59 of 67 cases (88.1%), corresponding to substantial agreement between ctDNA and imaging (Cohen’s κ = 0.755; 95% CI, 0.598-0.913). Using radiologic assessment as the reference standard, ctDNA demonstrated a sensitivity of 80.0% and specificity of 94.6% for detecting progressive disease. These assessment-level estimates were descriptive and did not account for repeated measurements within patients. Descriptive review of the longitudinal ctDNA identified patterns consistent with radiologic and clinical disease course in individual patients. In a 3-month landmark analysis, patients with ctDNA-defined progression had numerically shorter progression-free survival than patients without ctDNA-defined progression (9.0 vs. 16.0 months), although the difference was not statistically significant. Exploratory whole-genome MRD analysis was concordant with radiologic disease course in two selected patients.

**Conclusions:**

Tumor-informed ctDNA monitoring was feasible in 70% of screened patients and showed high concordance with radiologic disease assessment in this real-world sarcoma cohort. These findings support prospective evaluation of ctDNA as an adjunct to imaging for longitudinal disease monitoring in patients with a suitable tumor-informed target, while broader tumor-informed approaches may be required for patients whose tumors are not adequately covered by predefined targeted panels.

**Supplementary Information:**

The online version contains supplementary material available at https://doi.org/10.1186/s12885-026-17180-y.

## Background

Sarcomas comprise a rare and biologically heterogeneous group of malignant bone and soft tissue tumors with diverse molecular drivers, anatomical presentations, treatment strategies, and clinical trajectories [1]. Due to their rarity, the diagnosis and treatment of patients with sarcoma should be performed at, or in close collaboration with, specialized and certified sarcoma centers [2–7]. Despite advances in multimodal therapy, many patients develop recurrent or metastatic disease, and reliable blood-based biomarkers for longitudinal disease monitoring remain unavailable for most sarcoma subtypes [2, 3].

Current surveillance relies primarily on cross-sectional imaging. Although imaging remains indispensable, it has important limitations, including fixed surveillance intervals, limited sensitivity for early biological progression, and interpretative challenges in postoperative or post-radiation settings. These limitations are particularly relevant in anatomically complex sites, such as the pelvis and retroperitoneum, where viable tumor may be difficult to distinguish from fibrosis, necrosis, inflammation, or other treatment-related changes [3].

Circulating tumor DNA (ctDNA) analysis has emerged as a minimally invasive approach for assessing tumor burden, molecular response, molecular residual disease, and recurrence risk in solid tumors [8]. However, the clinical translation of ctDNA monitoring into sarcoma care is more challenging than in common epithelial malignancies. Sarcomas include translocation-driven entities, copy-number-altered sarcomas, and tumors with complex karyotypes, many of which lack recurrent hotspot mutations that can be captured by universal plasma-based assays [9]. Consequently, tumor-informed approaches are often required to enable longitudinal ctDNA monitoring in individual patients.

Several sarcoma-specific studies support the feasibility of ctDNA detection, while also demonstrating substantial heterogeneity in assay performance across histologic entities and clinical settings. Eastley et al. detected circulating tumor-derived DNA in patients with metastatic soft tissue sarcoma and subsequently characterized ctDNA in non-metastatic soft tissue sarcoma, highlighting the influence of disease burden and clinical context on detectability [10, 11]. Tsoi et al. evaluated the detection and potential utility of cell-free and circulating tumor DNA across bone and soft tissue sarcomas using personalized approaches [12]. Entity-specific studies have further shown that highly personalized ctDNA assays can detect disease burden in Ewing sarcoma and that digital PCR-based ctDNA analysis may support diagnosis, prognostication, and residual disease detection in chondrosarcoma [13, 14]. Across broader soft tissue sarcoma subtypes, Demoret et al. evaluated concordance between commercial ctDNA profiling and tumor-based sequencing, emphasizing both the feasibility and limitations of plasma-based molecular profiling in this heterogeneous disease group [15].

More recent sarcoma-focused studies have further strengthened the rationale for tumor-informed monitoring. Darville-O’Quinn et al. demonstrated that personalized assays based on tumor-specific variants can detect ctDNA in plasma samples from patients with sarcoma and may support longitudinal monitoring of disease status [16]. Sun et al. reported a retrospective cohort of 45 patients with soft tissue sarcoma in whom tumor-informed ctDNA dynamics were associated with treatment response and recurrence, supporting ctDNA as a potential adjunct to imaging-based surveillance in selected patients [17]. In high-grade osteosarcoma, Audinot et al. showed that ctDNA quantification provided additional prognostic information in a translational study from the OS2006 trial, further supporting the prognostic relevance of ctDNA in selected bone sarcoma settings [18].

Nevertheless, real-world data remain limited, and direct comparisons between longitudinal ctDNA findings and routine radiologic assessment in heterogeneous sarcoma cohorts are still scarce. In this retrospective real-world study, we evaluated tumor-informed ctDNA monitoring in patients with bone and soft tissue sarcomas treated at a certified high-volume sarcoma center. We assessed the proportion of screened patients with a suitable panel-covered tumor-specific alteration, analyzed longitudinal ctDNA dynamics, and compared ctDNA findings with paired radiologic assessments. In two selected patients, we additionally explored whole-genome–based MRD fingerprinting as a complementary approach for longitudinal disease monitoring.

## Methods

### Study design and patient cohort

We conducted a retrospective real-world study of patients with bone and soft tissue sarcomas treated at the certified high-volume sarcoma center of the University Hospital Tübingen, Germany. Patients were screened for eligibility if tumor molecular profiling and longitudinal clinical follow-up data were available. The study cohort comprised patients in whom tumor-informed ctDNA monitoring was technically feasible based on the identification of at least one suitable tumor-specific alteration covered by the predefined ctDNA monitoring panel.

A total of 20 patients were retrospectively identified and screened from our certified sarcoma center based on predefined eligibility criteria, including the availability of tumor tissue for paired tumor-normal sequencing and longitudinal plasma samples. Patients without sufficient tumor material or without longitudinal plasma samples could not be included. Fourteen patients (70%) had an identifiable tumor-informed ctDNA target and were included in the longitudinal analysis. Six patients were excluded because no suitable target for panel-based ctDNA monitoring was available. Clinical data, treatment history, radiologic findings, liquid biopsy results, and survival outcomes were extracted from electronic medical records. Treatment characteristics of the longitudinal ctDNA monitoring cohort, including systemic antitumor therapy, radiotherapy courses, and surgical procedures, are summarized in Supplementary Table S1. The data cutoff was October 15, 2025. Patient-level characteristics of all 20 screened patients, including histology, disease stage, baseline plasma sample availability, and eligibility for ctDNA monitoring, are provided in Supplementary Table S2. For the 14 patients included in longitudinal monitoring, this table additionally summarizes ctDNA detection, sample counts, concordant and discordant assessments, and treatment modalities during monitoring.

### Tumor molecular profiling and ctDNA target identification

Somatic molecular diagnostics were performed to identify single-nucleotide variants, small insertions and deletions, copy-number alterations, and gene fusions. Molecular profiling was carried out either by targeted tumor panel sequencing or whole-exome sequencing with matched tumor-normal analysis. Formalin-fixed paraffin-embedded tumor tissue was used as tumor material, and matched EDTA blood was used as the germline control. Sequencing was performed on Illumina platforms.

Patients were considered eligible for longitudinal ctDNA monitoring if at least one tumor-specific variant was identified within a genomic region covered by the highly sensitive UMI-based hotspot panel used for plasma monitoring. In these patients, serial blood samples were collected in stabilizing tubes, specifically Streck Cell-Free DNA BCT tubes, for longitudinal monitoring of the respective tumor-specific variants in cell-free DNA.

The diagnostic procedure and bioinformatic workflow have been described previously by Forschner et al. [19]. Initial somatic diagnostics in tumour-normal comparisons were carried out using large gene panels or at the exome level. Technically, this approach is fairly broad in scope, as it analyses the entire coding sequence of the target genes. Variants with a VAF (Variant Allele Fraction) of down to 5% can be detected in tumour tissue (Formalin-fixed paraffin-embedded, FFPE) and blood. For monitoring using cell free DNA (cfDNA), however, a UMI-based hotspot panel that is significantly more focused in the target region is used, in order to detect mutations with greater sensitivity by using higher sequencing depth. As the target region analysed differs between the UMI panel and standard Next Generation Sequencing (NGS) approaches, only patients whose mutations are present in the target region of both technical approaches can be included in the monitoring programme. For all sequencing approaches used, a minimum of 20 ng of DNA is required as starting material.

The UMI-based monitoring was performed using two versions of a custom-designed hybrid capture target enrichment panel (CFD.1 and CFD.2), designed by CeGaT and manufactured by Twist Biosciences. Twist Duplex UMI adapters (5 + 5 bases for UMI, 1024 combinations) are used in the library preparation (Twist Mechanical Fragmentation Protocol). Starting from 20ng cfDNA, library preparation and target enrichment were performed as per manufacturer’s instructions. Samples were sequenced to at least 15 Gbp (150m raw reads). Variant calling was restricted to SNVs and small indels with full duplex support. The assay can detect sequence variants with a minimum read support of 0.1% (e.g., 1 molecule in 1000) and was validated to have a sensitivity of 95% at 0.5% variant allele frequency (LOD, limit of detection). Laboratory personnel work on barcoded, de-identified samples and have no access to or knowledge of any patient information. The genes, reference transcripts, and target regions of the UMI-based ctDNA monitoring panel are listed in Supplementary Table S5.

For selected patients with a sufficient number of plasma sampling time points during the course of disease, tumor content in cfDNA was additionally assessed using a whole-genome-based molecular residual disease approach, referred to as MRD fingerprinting, in addition to UMI panel-based monitoring.

### Longitudinal ctDNA monitoring

For patients with an identifiable tumor-informed ctDNA target, serial plasma samples were obtained during routine clinical care. ctDNA monitoring was performed using a highly sensitive UMI-based targeted sequencing approach designed to track patient-specific tumor variants over time in plasma cfDNA.

For targeted longitudinal ctDNA monitoring, a plasma sample was classified as ctDNA-positive if the patient-specific tumor variant was detected above the validated assay-specific calling threshold after UMI-based error suppression and internal quality filtering. Samples in which the patient-specific variant was not detected above this threshold were classified as ctDNA-negative.

Conversion from ctDNA-positive to ctDNA-negative status was defined as loss of detectability of a previously detected patient-specific tumor variant in a subsequent plasma sample. Re-emergence was defined as renewed detection of the same patient-specific variant after at least one intervening ctDNA-negative sample. Increases and decreases in ctDNA were assessed relative to the laboratory-reported quantitative ctDNA measure in the preceding evaluable plasma sample from the same patient.

### Whole-genome MRD fingerprint generation

For MRD fingerprint generation, DNA was extracted from formalin-fixed paraffin-embedded tumor material, and matched normal DNA was extracted from EDTA blood. Sequencing libraries were constructed using the Twist Mechanical Fragmentation protocol (Twist Biosciences, USA), with fragmentation performed on a Covaris instrument (PerkinElmer, USA). Libraries were sequenced on a NovaSeq X+ instrument (Illumina, USA) with 2 × 100 bp paired-end reads, targeting at least 120 Gb of sequencing data per sample.

Raw sequencing data were demultiplexed using bcl2fastq, and adapter trimming was performed using Skewer. Reads were aligned to the human reference genome GRCh37, and somatic sequence variants were called using DRAGEN version 4.2 (Illumina, USA). Whole-genome sequencing achieved a sequencing depth of approximately 30-40x coverage.

Somatic variants were filtered to generate the final patient-specific MRD fingerprint. Variants were excluded if they met any of the following criteria: sequencing coverage below 10x in the tumor sample, sequencing coverage below 10x in the matched normal sample, evidence of the variant in the matched normal alignment below the formal variant-calling threshold, likely formalin-fixation artefact, indel classification, presence in gnomAD, or evidence suggesting subclonality based on a variant allele frequency that was markedly lower than the overall tumor variant allele frequency distribution.

### MRD tumor detection

For MRD detection, blood plasma was collected in stabilizing tubes, and cfDNA was extracted according to established procedures. Sequencing libraries were constructed as described above, except that no additional fragmentation step was performed. Sequencing, demultiplexing, adapter trimming, and read alignment were performed as described for MRD fingerprint generation, again targeting a sequencing depth of approximately 30-40x coverage per base.

The precomputed patient-specific MRD fingerprint was applied to the cfDNA sequencing data. For each fingerprint position, the cfDNA alignment was assessed for reads supporting the corresponding tumor-specific variant, yielding the number of detectable fingerprint variants.

To estimate the technical background signal, 100 random genomic positions were selected for each fingerprint variant. These background positions were matched for trinucleotide sequence context and were required to contain no germline variant in the corresponding patient. Background positions were then evaluated for reads supporting the same base change as the associated fingerprint variant, generating a distribution of background counts.

Tumor content was estimated by comparing the number of detectable fingerprint variants with the background distribution. The final tumor content estimate was corrected by subtracting the median background signal. The background distribution was also used to estimate the probability that the observed number of detectable fingerprint variants could be explained by technical error alone. A sample was classified as tumor-positive if this probability was below 0.01.

Based on internal validation, this whole-genome MRD approach can detect tumor fractions of approximately 0.05% when a patient-specific fingerprint comprising at least 1,000 variants is available; analytical sensitivity increases with fingerprint size.

### Radiologic assessment and clinical endpoints

Radiologic disease assessment was performed as part of routine clinical care using cross-sectional imaging. Imaging studies were classified according to the documented interpretation in the routine radiology report and the treating physicians’ clinical assessment. No blinded central radiologic review or uniform retrospective RECIST reassessment was performed. For the purpose of this analysis, disease status was categorized as progressive disease or non-progressive disease. Non-progressive disease included stable disease, partial response, complete response, or absence of radiologic evidence of disease, depending on the clinical context.

Liquid biopsy samples were paired one-to-one with corresponding radiologic assessments obtained within the same clinical assessment interval. The clinical assessment interval was defined as the period surrounding a routine radiologic evaluation during which treatment status and the treating physicians’ clinical assessment of disease status were considered unchanged. Each of the 67 liquid biopsy samples was matched to a distinct radiologic assessment; no radiologic assessment was used for more than one liquid biopsy sample. Paired assessments were classified as concordant when both modalities indicated either progressive disease or non-progressive disease and as discordant when the classifications differed. For each paired assessment, the sampling-imaging interval was calculated as the date of blood collection minus the acquisition date of the paired radiological examination. Negative values indicate blood collection before imaging, positive values indicate blood collection after imaging, and a value of zero indicates same-day assessments. Intervals were summarized using medians and ranges while retaining their signs. Individual intervals and patient-level summaries are provided in Supplementary Table S3. Discordant assessments were reviewed individually with respect to histology, disease stage and sites, the sampling-imaging interval, treatment exposure, ctDNA findings, and subsequent imaging. These findings and possible explanations for discordance are summarized in Supplementary Table S4. Proposed explanations were considered descriptive interpretations rather than established causes.

Progression-free survival was defined as the time from initial sarcoma diagnosis to the first documented radiologic or clinical disease progression or death from any cause, whichever occurred first. This endpoint was used descriptively to characterize the overall disease course of the cohort and was not intended to represent treatment-line-specific PFS or PFS from initiation of ctDNA monitoring. Overall survival was defined as the time from initial sarcoma diagnosis to death from any cause. Patients without the respective event were censored at the date of last clinical follow-up. Descriptive Kaplan-Meier estimates of progression-free and overall survival from initial sarcoma diagnosis are presented in Supplementary Figures S1 and S2, respectively.

For the 3-month landmark analysis, patients were included only if they were alive, progression-free at the landmark time point, and had evaluable ctDNA data. Patients were classified according to ctDNA status at the 3-month landmark, and subsequent PFS was calculated from the landmark date to progression or death.

### Statistical analysis

Descriptive statistics were used to summarize patient characteristics, treatment modalities, ctDNA target availability, liquid biopsy findings, and clinical outcomes. Continuous variables are presented as medians with ranges or means, as appropriate. Sampling-imaging intervals were summarized both per patient and across all paired assessments, retaining their signs. The overall median was calculated from all individual paired intervals rather than from patient-specific medians. The numbers and percentages of blood samples collected before, on the same day as, or after the paired imaging examination were also calculated. Categorical variables are presented as absolute numbers and percentages.

Agreement between liquid biopsy and radiologic assessment was evaluated using Cohen’s kappa. Sensitivity and specificity of liquid biopsy for the detection of progressive disease were calculated using radiologic assessment as the reference standard. An unadjusted association between liquid biopsy classification and radiologic disease status was assessed using Fisher’s exact test, and the corresponding odds ratio was reported. These estimates did not account for within-patient clustering and were therefore considered exploratory.

Progression-free survival and overall survival were estimated using the Kaplan–Meier method. A 3-month landmark analysis was performed in patients who were progression-free at 3 months and had evaluable ctDNA data. Survival distributions were compared using the log-rank test. Cox proportional hazards regression was used to estimate hazard ratios with 95% confidence intervals.

Because multiple paired liquid biopsy–radiologic assessments could originate from the same patient, paired observations were not statistically independent. The number of paired assessments per patient was summarized descriptively. Given the small cohort size, no formal cluster-adjusted model was performed. Agreement, sensitivity, specificity, odds ratio, and Fisher’s exact test results were therefore interpreted as descriptive and hypothesis-generating rather than confirmatory.

Statistical analyses were performed using GraphPad Prism version 10.6.1 (892). All p values were two-sided and are reported descriptively without adjustment for multiple testing. Given the small sample size and exploratory nature of the study, all analyses were considered hypothesis-generating and descriptive rather than confirmatory.

### Ethics approval

This study was conducted in accordance with the Declaration of Helsinki and was approved by the Ethics Committee of the Faculty of Medicine of the University of Tübingen and the University Hospital Tübingen, Germany (approval number 194/2025BO2). All patients had provided the University Hospital Tübingen Biomaterial Broad Consent for the use of biomaterial and associated clinical data for research purposes. The requirement for additional study-specific written informed consent for this retrospective analysis of pseudonymized data was waived by the ethics committee.

## Results

### Study cohort and ctDNA target identification

A total of 20 patients with bone or soft tissue sarcoma were screened for tumor-informed ctDNA monitoring. The screened cohort included 13 male and 7 female patients, with a mean age of 40.9 years, a median age of 36.0 years, and an age range of 20–65 years. Fourteen patients (70%) met the molecular inclusion criteria for longitudinal ctDNA monitoring, whereas six patients (30.0%) had no suitable panel-covered tumor-specific alteration for panel-based ctDNA monitoring.

The longitudinal ctDNA monitoring cohort included 11 male and 3 female patients, with a mean age of 39.9 years, a median age of 31.0 years, and an age range of 20–65 years. Ewing sarcoma represented the largest histologic subgroup in the longitudinal ctDNA monitoring cohort (7/14, 50.0%). The cohort was enriched for high-grade disease, with grade 3 tumors documented in 11 of 13 patients with known tumor grade (84.6%). UICC (Union for International Cancer Control) stage III or IV disease was present in 8 of 14 patients (57.1%) in the longitudinal ctDNA monitoring cohort and in 14 of 20 patients (70.0%) in the screened cohort. Demographic and histopathological characteristics of the longitudinal ctDNA monitoring cohort are summarized in Tables 1 and 2. Individual data for all 20 screened patients, including those without a suitable monitoring target, are provided in Supplementary Table S2.

**Table 1:** Demographic, molecular eligibility, and histologic characteristics

Characteristic	Longitudinal ctDNA monitoring cohort, n = 14
Demographic characteristics	
Age, mean ± SD, years	39.9 ± 17.8
Age, median (range), years	31.0 (20–65)
Male sex, n (%)	11 (78.6)
Female sex, n (%)	3 (21.4)
Molecular eligibility	
Suitable panel-covered tumor-specific alteration identified, n (%)	14 (100.0)
Histology	
Ewing sarcoma, n (%)	7 (50.0)
Desmoplastic small round cell tumor (DSRCT), n (%)	1 (7.1)
Leiomyosarcoma, n (%)	1 (7.1)
Myxoid pleomorphic liposarcoma, n (%)	1 (7.1)
Angiosarcoma, n (%)	1 (7.1)
Malignant peripheral nerve sheath tumor (MPNST), n (%)	1 (7.1)
Myxoid liposarcoma, n (%)	1 (7.1)
Embryonal rhabdomyosarcoma, n (%)	1 (7.1)

Data are presented as n (%) unless otherwise indicated. All percentages were calculated using the full longitudinal ctDNA monitoring cohort of 14 patients

**Table 2:** UICC stage and tumor grade

Characteristic	Longitudinal ctDNA monitoring cohort, n ﻿= 14
UICC stage at diagnosis	
UICC I, n (%)	0 (0.0)
UICC II, n (%)	6 (42.9)
UICC III, n (%)	2 (14.3)
UICC IV, n (%)	6 (42.9)
Advanced-stage disease, UICC III–IV, n (%)	8 (57.1)
Histological tumor grading according to FNCLCC/WHO	
Grade 1, n (%)	1 (7.1)
Grade 2, n (%)	1 (7.1)
Grade 3, n (%)	11 (78.6)
Grade not available, n (%)	1 (7.1)
Grade 3 among patients with known tumor grade, n/N (%)	11/13 (84.6)

Percentages for individual tumor-grade categories were calculated using the full cohort of 14 patients. The proportion of grade 3 tumors among patients with known tumor grade was calculated using 13 patients as the denominator.

### Treatment characteristics

Complete treatment data were available for all 14 patients in the longitudinal ctDNA monitoring cohort. All patients received systemic antitumor therapy during their disease course, with heterogeneous regimens and frequently multiple treatment lines. Radiotherapy was administered to 10 patients (71.4%), comprising 13 courses, and 12 patients (85.7%) underwent a total of 16 surgical procedures. Treatment characteristics, including the distribution of radiotherapy and surgery between primary-tumor and metastatic settings, are summarized in Supplementary Table S1. Treatment modalities during ctDNA monitoring are provided at the patient level in Supplementary Table S2.

Radiotherapy was administered to 10 of 14 patients (71.4%), accounting for a total of 13 radiotherapy courses. Radiotherapy was delivered exclusively for metastatic disease in four patients (28.6%), in both primary-tumor and metastatic settings in three patients (21.4%), and exclusively in the primary-tumor setting in three patients (21.4%).

Surgical treatment was performed in 12 of 14 patients (85.7%), accounting for a total of 16 surgical procedures. Surgery was performed exclusively in the primary-tumor setting in six patients (42.9%), exclusively for metastatic disease in two patients (14.3%), and in both primary-tumor and metastatic settings in four patients (28.6%). Treatment characteristics are summarized in Supplementary Table S1.

### Survival outcomes

Kaplan–Meier estimates of progression-free survival and overall survival from initial sarcoma diagnosis are shown separately in Supplementary Figures S1 and S2, respectively. In the screened cohort of 20 patients, Kaplan-Meier estimates of median progression-free survival and overall survival from initial sarcoma diagnosis were 12.0 and 26.0 months, respectively. Nineteen patients experienced progression during follow-up. One patient remained alive and progression-free and was censored at 44.4 months. These analyses were descriptive because patients entered ctDNA monitoring at different stages of their disease course and survival was not assessed from a uniform treatment or molecular-monitoring baseline.

### Longitudinal ctDNA monitoring

All patients included in the final analysis had an identifiable tumor-informed ctDNA target and underwent longitudinal plasma-based ctDNA monitoring during routine clinical care (for details see Table 3). A total of 67 longitudinal plasma samples were analyzed across the 14 included patients, with a median of 4 samples per patient (range: 2-10).

**Table 3:** Longitudinal ctDNA monitoring characteristics

Characteristic	Longitudinal ctDNA monitoring cohort, n﻿ = 14
Longitudinal plasma samples, total n	67
Plasma samples per patient, median (range)	4 (2-10)
Paired liquid biopsy-radiologic assessments, total n	67
Paired assessments per patient, median (range)	4 (2-10)

Each liquid biopsy sample was paired with a distinct radiologic assessment. Repeated assessments were clustered within individual patients and are therefore reported descriptively.

ctDNA dynamics varied between patients and across time points, consistent with the biological and clinical heterogeneity of sarcoma and known variability in ctDNA shedding. Descriptive review of the longitudinal trajectories identified changes in ctDNA detectability and quantitative signal that were consistent with clinical disease activity in individual patients. Decreasing ctDNA signals or conversion from ctDNA-positive to ctDNA-negative status were observed in association with treatment response or disease control, whereas re-emergence or increasing ctDNA signals were observed in association with radiologic or clinical disease progression. These patterns were not consistent across all assessments, as reflected by the discordant cases detailed in Supplementary Table S4.

### Concordance between liquid biopsy and radiologic assessment

A total of 67 paired liquid biopsy-radiologic assessments were analyzed, with a median of 4 paired assessments per patient (range: 2-10). ctDNA was detected at least once during monitoring in all 14 patients with a suitable target, although detection was not consistent across all sampling time points (Supplementary Table S2). Paired assessments were considered evaluable when liquid biopsy and imaging were available within the same clinical assessment interval. The median signed sampling-imaging interval was + 2 days (range: -20 to +34 days). Blood collection preceded imaging in 21 assessments (31.3%), occurred on the same day in 12 (17.9%), and followed imaging in 34 (50.7%). Individual intervals and patient-level summaries are provided in Supplementary Table S3.

Concordant assessments were observed in 59 of 67 cases (88.1%). Agreement between liquid biopsy and radiologic assessment was substantial, with a Cohen’s kappa of 0.755 (95% CI, 0.598-0.913). Using radiologic assessment as the reference standard, liquid biopsy demonstrated a sensitivity of 80.0% and a specificity of 94.6% for the detection of progressive disease.

**Fig. 1:**
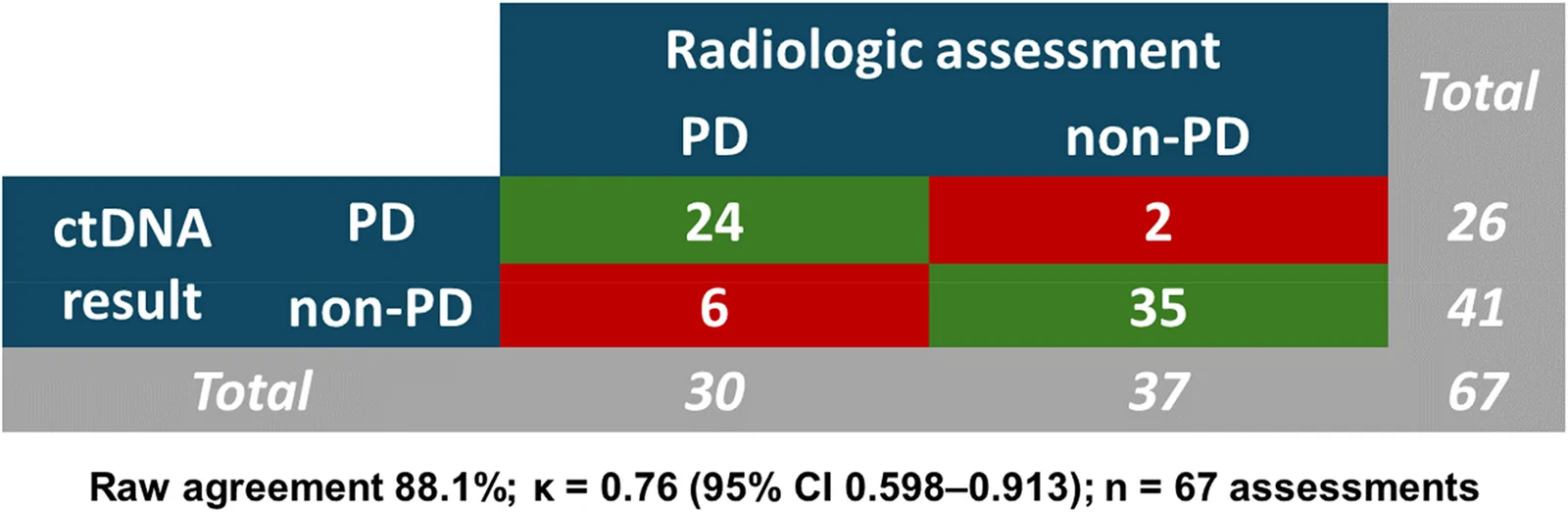
Concordance between ctDNA and radiologic disease assessment. A total of 67 paired assessments were analyzed. Concordant results were observed in 59 of 67 assessments (88.1%). Cohen’s κ was 0.755 (95% CI, 0.598–0.913). The confidence interval for Cohen’s κ was calculated without adjustment for within-patient clustering. Using radiologic assessment as the reference standard, ctDNA demonstrated a sensitivity of 80.0% and specificity of 94.6% for detecting progressive disease. In an unadjusted analysis that did not account for within-patient clustering, the odds ratio was 70.0 and Fisher’s exact p value was <0.0001. These finding should be interpreted as descriptive and hypothesis-generating rather than confirmatory because repeated assessments from individual patients were not statistically independent

In an unadjusted analysis that did not account for within-patient clustering, the odds ratio for the association between liquid biopsy classification and radiologic disease status was 70.0, and Fisher’s exact p value was < 0.0001. Because repeated assessments within individual patients were not statistically independent and no adjustment for within-patient clustering was performed, the odds ratio and Fisher’s exact test p value are reported descriptively and should not be interpreted as confirmatory statistical evidence. The complete concordance matrix, including marginal totals, is shown in Fig. 1. Eight assessments were discordant: six showed radiological progression without ctDNA-defined progression, whereas two showed ctDNA-defined progression without radiological progression at the paired assessment. The eight discordant assessments occurred in five of the 14 monitored patients. The remaining nine patients had concordant classifications at all paired assessments (Supplementary Table S2). Case-level details, including disease characteristics, sampling-imaging intervals, treatment exposure, ctDNA findings, subsequent imaging, and possible explanations for discordance, are provided in Supplementary Table S4. The cause of discordance could not be established in all cases.

### Three-month landmark analysis

A 3-month landmark analysis was performed in patients who were alive and progression-free at 3 months and had evaluable ctDNA data. Patients were classified according to ctDNA status at the landmark, and progression-free survival was assessed from the landmark date onward. Median progression-free survival measured from the landmark date was numerically shorter in patients with ctDNA-defined progression than in those without ctDNA-defined progression (9.0 versus 16.0 months, respectively).

This difference did not reach statistical significance (log-rank *p* = 0.2109). Cox proportional hazards regression yielded a hazard ratio of 2.39 (95% CI, 0.29-19.87). The estimate was imprecise, and the analysis was underpowered because of the small number of eligible patients and progression events. The findings should therefore be interpreted as exploratory and hypothesis-generating.

### Exploratory whole-genome MRD analysis

Longitudinal whole-genome-based MRD data were available for two selected patients with metastatic Ewing sarcoma and were analyzed descriptively.

Patient-specific MRD fingerprints were generated from tumor-normal whole-genome sequencing and applied to longitudinal cfDNA samples.

**Fig. 2:**
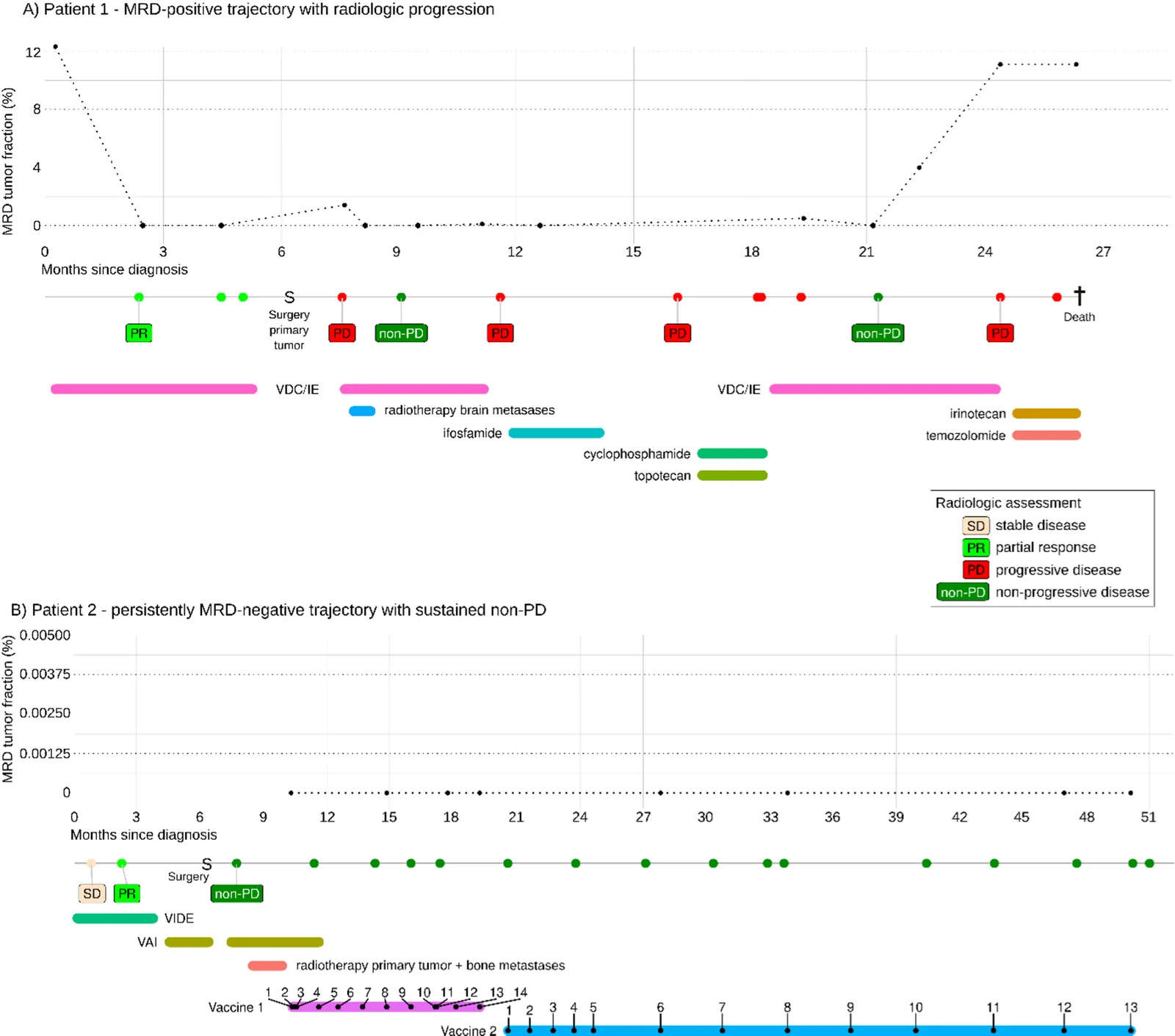
Longitudinal whole-genome MRD trajectories in two selected patients. **Panel A **shows an MRD-positive trajectory associated with radiologic progression, whereas **Panel B** shows a persistently MRD-negative trajectory associated with sustained radiologic non-progression. Panels** A** and **B** display patient-specific whole-genome MRD tumor fraction over time in relation to radiologic disease assessment, treatment exposure, and clinical course. Radiologic assessments are shown as progressive disease (PD) or non-progressive disease (non-PD), consistent with the binary classification used in the concordance analysis. Where applicable, the underlying clinical response category is shown in parentheses. Different y-axis scales were used in Panels **A** and **B**. In Panel **B**, all MRD measurements remained below the assay-defined positivity threshold. The patient-specific fingerprint in Panel **B** comprised 419 variants, with a reported limit of detection of 0.15% tumor fraction. Lines connecting MRD measurements are shown for visualization only

In one patient, MRD positivity coincided with radiologic progression, whereas MRD negativity was associated with treatment response. Changes in MRD levels were observed in relation to the radiologic disease course. Their temporal relationship to imaging and treatment is shown in Fig. 2. In the second patient, MRD remained below the assay-specific positivity threshold throughout the observed follow-up, while serial imaging showed no radiological progression.

The two cases illustrate longitudinal whole-genome-based ctDNA monitoring during radiological progression and non-progression, respectively. Persistently negative findings in the second patient do not establish assay sensitivity in that individual. However, because only two selected patients were analyzed, these observations do not permit estimation of diagnostic sensitivity, specificity, prognostic performance, or clinical utility. Longitudinal MRD trajectories in relation to radiologic assessment, treatment exposure, and clinical course are shown in Fig. 2.

## Discussion

In this retrospective real-world study, a suitable target for panel-based ctDNA monitoring was identified in 14 of 20 screened patients with bone and soft tissue sarcoma. In these 14 patients, longitudinal ctDNA monitoring showed high observed agreement with radiological progression classification. Among 67 paired liquid biopsy–radiologic assessments, both modalities were concordant in 88.1% of cases, corresponding to substantial agreement by Cohen’s kappa. Using radiologic assessment as the reference standard, ctDNA demonstrated a sensitivity of 80.0% and a specificity of 94.6% for detecting progressive disease. These findings suggest that tumor-informed ctDNA may serve as an adjunct to radiologic assessment in selected patients with a suitable panel-covered tumor-specific alteration. However, the results should be interpreted cautiously because repeated assessments were clustered within individual patients and no cluster-adjusted analysis was performed.

Descriptive review of longitudinal ctDNA trajectories identified patterns consistent with clinical disease activity in individual patients. Decreasing ctDNA signals or conversion from detectable to undetectable ctDNA were observed in association with treatment response or disease control, whereas renewed detection or increasing ctDNA signals were associated with radiologic or clinical progression. These observations suggest that ctDNA dynamics may reflect changes in disease activity, although a quantitative relationship with radiological tumor burden was not established in this study. In contrast to imaging, which is usually performed at predefined intervals and may be difficult to interpret after surgery, radiotherapy, or multimodal treatment, ctDNA can be sampled repeatedly and may provide temporally complementary information on disease. Whether ctDNA changes can guide the timing of additional imaging requires prospective evaluation. The present analysis does not establish a molecular lead time over imaging, as it primarily assessed concordance between paired examinations rather than systematically evaluating the interval from molecular to radiological progression. The analysis compared paired ctDNA and radiological classifications but did not formally model ctDNA trajectories across consecutive imaging intervals. Future studies should evaluate molecular and radiological changes over corresponding intervals using standardized sampling schedules.

The observed agreement between ctDNA and imaging is clinically relevant. The observed specificity of 94.6% indicates that ctDNA-defined progression was uncommon among assessments classified as radiological non-progression. This estimate relates to the binary progression classification and should not be interpreted as the specificity of ctDNA detection for the presence of sarcoma. Detection of a known tumor-specific variant supports the presence of tumor-derived DNA but does not by itself establish disease progression. A ctDNA-PD classification discordant with radiologic non-progression should therefore not automatically be interpreted as an analytically false-positive result; such findings require clinical contextualization to avoid unnecessary patient anxiety, intensified surveillance, or premature treatment changes. However, ctDNA-defined progression was identified in only 24 of the 30 assessments classified as radiological progression, corresponding to an observed sensitivity of 80.0%. The six discordant assessments demonstrate that a ctDNA non-PD classification did not exclude radiological progression (Supplementary Table S4). Potential explanations include low or variable ctDNA shedding, limited tumor burden, anatomical factors, intratumoral heterogeneity, insufficient cfDNA input, or underrepresentation of the tracked tumor clone. At present, therefore, ctDNA should not be considered a replacement for conventional imaging. Rather, it may be most useful as an adjunctive tool in patients with an identifiable tumor-informed target and documented ctDNA detectability during the disease course.

The feasibility rate of 70% is another important finding. Sarcomas are biologically and genomically heterogeneous and may lack recurrent hotspot alterations suitable for predefined plasma-based assays. Target availability depends on tumor subtype, tissue quality, sequencing strategy, tumor purity, assay design, and whether the detected tumor-specific alteration lies within the target region of the monitoring panel. The 70% feasibility rate therefore primarily reflects the applicability of the predefined monitoring panel rather than the biological presence or absence of ctDNA. Patients without a suitable target may still shed ctDNA but may harbor alterations, including oncogenic gene fusions, that are not covered or cannot be adequately tracked by the targeted assay. Accordingly, the absence of a suitable target should not be interpreted as evidence of absent ctDNA shedding or less aggressive tumor biology. Broader tumor-informed approaches that use larger patient-specific variant fingerprints may reduce this limitation, although their analytical and clinical performance in sarcoma requires prospective validation.

The 3-month landmark analysis showed numerically shorter PFS in patients with ctDNA-defined progression, although the difference was not statistically significant and the confidence interval was wide. The analysis was underpowered because of the small number of eligible patients and progression events, and the resulting effect estimate was imprecise. The direction of the association is consistent with observations from other solid tumors, in which persistent or increasing ctDNA and postoperative molecular residual disease have been associated with recurrence and adverse outcomes. Nevertheless, sarcoma-specific validation remains necessary because ctDNA shedding, genomic architecture, and assay sensitivity differ substantially across histologic subtypes. Prospective studies with standardized sampling, a prespecified landmark definition and uniform clinical assessment time points are therefore required to quantify the prognostic performance of early ctDNA changes in sarcoma.

The exploratory whole-genome MRD analysis illustrates the potential value of broader tumor-informed approaches in sarcoma. In two selected patients with extended longitudinal follow-up, MRD findings were concordant with the radiologic disease course. In one patient, MRD positivity and increasing tumor fraction were associated with radiologic progression, whereas negative findings were observed during treatment response. In the second patient, persistently negative MRD findings accompanied an absence of radiological progression. No baseline plasma sample was available for whole-genome-based MRD assessment in this patient. However, without a documented positive result from the same assay during a period of radiologically evident disease, these negative findings do not establish the assay’s sensitivity in this individual or exclude residual disease below its detection limit. These observations support the technical feasibility and biological plausibility of whole-genome MRD monitoring in sarcoma but should be interpreted strictly as proof of concept. Because only two selected patients were analyzed, the data do not permit estimation of diagnostic sensitivity, specificity, prognostic performance, or clinical utility.

Across several solid tumors, ctDNA-based MRD detection has been associated with recurrence risk and prognosis; however, clinical implementation remains limited by tumor-specific differences in evidence, assay performance, and the lack of standardized testing and management algorithms [17]. In sarcoma, a genome-wide tumor-informed approach may increase assay applicability by enabling the aggregation of multiple patient-specific somatic variants without requiring a predefined panel-covered alteration. This may be particularly relevant for fusion-driven tumors in which no suitable alteration is covered by the predefined monitoring panel. However, analytical sensitivity remains dependent on the number of informative fingerprint variants, cfDNA input, sequencing depth, background error characteristics, and tumor shedding. Consequently, small patient-specific fingerprints may result in lower analytical sensitivity, and broader genomic coverage should not be assumed to provide uniformly superior performance. Formal analytical validation and prospective clinical evaluation are therefore required. The dataset did not permit a systematic comparison of the targeted UMI-based assay and the whole-genome-based approach; therefore, no conclusion can be drawn regarding their relative sensitivity or clinical performance. Our findings are consistent with the growing sarcoma ctDNA literature but also address a distinct real-world question. Earlier studies have shown that ctDNA can be detected in metastatic and non-metastatic soft tissue sarcoma, although detectability varies by tumor burden and clinical setting [10, 11]. Personalized approaches have demonstrated technical feasibility and potential clinical utility across bone and soft tissue sarcomas, including Ewing sarcoma and chondrosarcoma, where tumor-informed or entity-specific assays may be particularly informative [12–14]. Commercial and tumor-informed assays have also shown associations with tumor sequencing, recurrence, treatment response, or clinical outcomes across selected sarcoma cohorts [15–18]. The present study extends this evidence by evaluating longitudinal tumor-informed ctDNA monitoring in a heterogeneous real-world cohort and by directly comparing serial ctDNA findings with routine radiologic disease assessments across multiple time points.

This study has several limitations. First, it was retrospective and conducted at a single tertiary sarcoma center, which may limit generalizability. Second, the cohort was small and histologically heterogeneous, with Ewing sarcoma accounting for half of the monitored patients. The pooled estimates therefore cannot establish comparable performance across sarcoma subtypes and require validation in larger, histologically homogeneous cohorts and an independent validation series. Third, liquid biopsy sampling and imaging intervals were not standardized but reflected routine clinical care. The signed sampling-imaging intervals ranged from -20 to +34 days, with blood collection occurring after imaging in approximately half of the assessments (Supplementary Table S3). Consequently, paired examinations did not necessarily reflect the same biological time point, which may have contributed to discordance. The median signed interval of +2 days should not be interpreted as indicating uniformly close temporal pairing. Fourth, due to the retrospective real-world design, standardized RECIST (Response Evaluation Criteria in Solid Tumors) assessments were not consistently available for all imaging examinations. We recommend prospective studies incorporating standardized RECIST or volumetric analyses together with serial ctDNA measurements. Fifth, only patients with an identifiable ctDNA target were included in the longitudinal ctDNA analysis, introducing an inherent selection bias. Sixth, paired assessments were clustered within individual patients, and several patients contributed multiple time points. Therefore, the 67 paired assessments should not be interpreted as fully independent observations. Patients contributed between two and ten paired assessments, so those with more assessments had a greater influence on the pooled agreement estimate. Because of the small cohort size, cluster-adjusted modelling was not performed, and agreement measures, sensitivity, specificity, and unadjusted association estimates should be interpreted descriptively. All statistical analyses should be considered hypothesis-generating and descriptive rather than confirmatory.

Seventh, PFS was calculated from initial diagnosis rather than from first liquid biopsy or the start of a specific treatment line. The descriptive PFS and OS analyses included all 20 screened patients, including the six without a suitable ctDNA monitoring target. These analyses characterize the disease course of the screened cohort and do not establish the prognostic value of ctDNA monitoring. The 3-month landmark analysis was additionally limited by the small number of eligible patients and events, resulting in imprecise effect estimates. Eighth, negative ctDNA findings may reflect limited tumor DNA shedding, low disease burden, or analytical sensitivity limitations rather than true biological absence of disease. Accordingly, a negative ctDNA result paired with radiological non-progression contributes to agreement in the binary PD/non-PD analysis but does not demonstrate that the assay reliably detected or excluded existing tumor burden. Thus, ctDNA should currently be interpreted as a complementary biomarker rather than a replacement for radiological imaging. Finally, the whole-genome-based MRD analysis was restricted to two patients selected for their extended longitudinal follow-up. These cases may not represent the broader cohort, and their findings should be considered exploratory. This MRD assay, as any assay, has a limit of detection (LOD). The MRD assay used for these patients was developed and validated using both an externally produced standard sample in a titration series, as well as artificial mixtures of patient tumor and normal DNA to assess assay performance and determine the LOD. As with all variant-based MRD assays, the LOD is dependent on the number of variants available for monitoring (the “fingerprint”), which in turn depends on the mutational load of the individual’s tumor. The patients monitored for this publication had very small fingerprints, concordant with the expected low mutational burden in sarcomas. For the clinically stable patient in this cohort, a fingerprint of 419 variants could be generated for MRD monitoring. According to the validation data, this allows detection of MRD positivity down to an LOD of 0.15% tumor content (1500 ppm), which is stated in all monitoring reports as well. A negative result does not exclude residual disease when the plasma tumor fraction is below this patient-specific detection limit.

Despite these limitations, the study has several strengths. It reflects routine clinical practice in a rare and heterogeneous tumor group, includes repeated longitudinal ctDNA assessments, transparently reports the proportion of screened patients with a suitable panel-covered tumor-specific alteration, provides the distribution of paired assessments per patient, and directly compares liquid biopsy findings with paired radiologic assessments. The one-to-one pairing of liquid biopsy samples with distinct radiologic examinations and the reporting of individual sampling–imaging intervals improve the transparency of the concordance analysis. However, this pairing does not eliminate the statistical dependence of repeated assessments within the same patient. This is particularly relevant in sarcoma, where technical feasibility, assay applicability, and longitudinal integration with routine imaging are themselves clinically important questions.

Taken together, these findings support the further development of tumor-informed ctDNA monitoring as a complementary tool for sarcoma surveillance and treatment monitoring. At present, ctDNA should be interpreted in the context of imaging, clinical findings, treatment status, and known assay limitations. Due to the hypothesis-generating nature of this study, our results require confirmation in future larger prospective multicenter studies with standardized imaging, longitudinal ctDNA assessments, standardized sampling schedules, predefined radiologic comparison intervals, broader tumor-informed assays, a patient-clustered analysis, a sensitivity analysis, and clinically meaningful decision points to determine the analytical validity, prognostic performance, and clinical utility of ctDNA monitoring in sarcoma. Ultimately, interventional studies will be required to establish whether ctDNA-guided management improves clinical decision-making or patient outcomes.

## Conclusions

In this retrospective real-world sarcoma cohort, a suitable target for panel-based ctDNA monitoring was identified in 70% of screened patients. Among these selected patients, paired ctDNA and radiological progression classifications showed an observed agreement of 88.1% during longitudinal follow-up. Descriptive review of longitudinal ctDNA trajectories identified patterns consistent with treatment response or disease progression in individual patients, supporting further evaluation of ctDNA as an adjunct to imaging.

These findings do not establish that ctDNA-guided management improves patient outcomes or that ctDNA monitoring can replace imaging. The applicability of predefined targeted assays remains limited by the availability of suitable panel-covered tumor variants, particularly in fusion-driven sarcomas. Broader genome-wide tumor-informed MRD approaches using large patient-specific variant fingerprints may reduce this limitation, but their analytical sensitivity, prognostic performance, and clinical utility in sarcoma require prospective validation. Variable ctDNA shedding, retrospective sampling, clustering of repeated assessments within individual patients, and the small cohort size remain additional limitations. Prospective studies with standardized sampling schedules, larger cohorts, broader tumor-informed approaches, and predefined clinical decision points are needed to determine whether ctDNA-guided monitoring can improve clinical decision-making and patient outcomes in bone and soft tissue sarcoma.

## Supplementary Information

Supplementary Material 1

## Data Availability

The datasets generated and/or analyzed during the current study are not publicly available due to patient privacy, data protection regulations, and the potentially identifiable nature of genomic and clinical data. Pseudonymized data may be made available from the corresponding author upon reasonable request and subject to approval by the responsible institutional authorities and applicable data protection regulations.

## Abbreviations

cfDNA: Cell-free DNA
CI: Confidence interval
ctDNA: Circulating tumor DNA
FFPE: Formalin-fixed paraffin-embedded
FNCLCC: Fédération Nationale des Centres de Lutte Contre le Cancer
I2VA: Ifosfamide, Vincristine, Actinomycin D
I2VAd: Ifosfamide, Vincristine, Doxorubicin
LOD: Limit of detection
MRD: Molecular residual disease
NGS: Next Generation Sequencing
OS: Overall survival
PD: Progressive disease
PFS: Progression-free survival
RECIST: Response Evaluation Criteria in Solid Tumors
UMI: Unique Molecular Identifier
UICC: Union for International Cancer Control
VAF: Variant Allele Fraction
VAI: Vincristine, Actinomycin D, Ifosfamide
VDC/IE: Vincristine, Doxorubicin, Cyclophosphamide alternating with Ifosfamide and Etoposide
VIDE: Vincristine, Ifosfamide, Doxorubicin, Etoposide
WES: Whole-exome sequencing
WGS: Whole-genome sequencing
WHO: World Health Organization
