# Supplementary material for: Association of tumor-informed circulating tumor DNA dynamics with radiologic disease assessment in a single-center real-world sarcoma cohort
Source: BMC Cancer. 2026 Oct 27;26:1311. doi: 10.1186/s12885-026-17180-y (PMC13715185; doi:10.1186/s12885-026-17180-y)
Supplement: Supplementary file 1 [file 12885_2026_17180_MOESM1_ESM.docx]

# Supplementary Material

#### **Supplementary Table S1: Treatment characteristics**

| Characteristic | Longitudinal ctDNA monitoring cohort, n = 14 |
| --- | --- |
| Systemic antitumor therapy |  |
| Systemic antitumor therapy, n (%) | 14 (100.0) |
| Radiotherapy |  |
| Patients receiving radiotherapy, n (%) | 10 (71.4) |
| Total radiotherapy courses, n | 13 |
| Radiotherapy exclusively for metastatic disease, n (%) | 4 (28.6) |
| Radiotherapy in both primary-tumor and metastatic  settings, n (%) | 3 (21.4) |
| Radiotherapy exclusively in the primary-tumor setting, n  (%) | 3 (21.4) |
| Surgery |  |
| Patients undergoing surgery, n (%) | 12 (85.7) |
| Total surgical procedures, n | 16 |
| Surgery exclusively in the primary-tumor setting, n (%) | 6 (42.9) |
| Surgery exclusively for metastatic disease, n (%) | 2 (14.3) |
| Surgery in both primary-tumor and metastatic settings, n  (%) | 4 (28.6) |

Footnote: Data are presented as n (%) unless otherwise indicated. All percentages were calculated using the longitudinal ctDNA monitoring cohort (n = 14) as the denominator. Patients could receive multiple treatment modalities and undergo more than one radiotherapy course or surgical procedure. Total numbers of courses and procedures are reported separately from patient counts. ctDNA, circulating tumor DNA.

#### **Supplementary Table S2: Patient-level Data**

| Patient | Histology | Disease Stage at monitoring initiation (UICC) | Baseline plasma sample | Baseline ctDNA status | Monitored genetic alterations | ctDNA detection | Number of longitudinal blood samples | Number of concordant (conc.) and disconcordant (disc.) assessments | Treatment during ctDNA monitoring |
| --- | --- | --- | --- | --- | --- | --- | --- | --- | --- |
| 1 | Ewing sarcoma | IV | yes | positive | EWSR1-FLI1, PIK3CA, PTEN, CDKN2A/B, BRAF | yes | 10 | 8/2 | 1,2 |
| 2 | Osseus Ewing sarcoma | IIB | yes | positive | CDKN2A | yes | 5 | 5/0 | 1 |
| 3 | Leiomyosarcoma | IV | yes | positive | PTEN, TP53, KIT | yes | 4 | 4/0 | 1,2,3 |
| 4 | Ewing sarcoma | IIB | no | NA | EWSR1-FLI1, TERT | yes | 3 | 2/1 | 1,2, |
| 5 | Osseus Ewing sarcoma | IIB | yes | positive | EWSR1-FLI1 | yes | 5 | 5/0 | 1 |
| 6 | Osseus Ewing sarcoma | IV | yes | positive | EWSR1-FLI1 | yes | 4 | 3/1 | 1 |
| 7 | Myxoid pleomorphic liposarcoma | IV | yes | positive | TP53 | yes | 4 | 4/0 | 1,2 |
| 8 | Osseus Ewing sarcoma | IIB | no | NA | TP53 (c.535), TP53 (c.776) | yes | 2 | 1/1 | 1 |
| 9 | Hepatic angiosarcoma | IIIA | yes | positive | TP53 (c.994), TP53 (c.770) | yes | 5 | 5/0 | 1,3 |
| 10 | Desmoplastic small roundcell tumor (DSRT) | IV | no | NA | EWSR1-WT1, STAG2, TP53 | yes | 7 | 4/3 | 1,3 |
| 11 | Osseus Ewing sarcoma | IVb | yes | negative | TP53 | yes | 9 | 9/0 | 1,2,3 |
| 12 | Maligne peripheral nerve sheath tumor (MPNST) | III | yes | negative | TERT | yes | 3 | 3/0 | 1,3 |
| 13 | Myxoid liposarcoma | IV | yes | positive | TERT, PIK3CA | yes | 4 | 4/0 | 1 |
| 14 | Embryonal rhabdomyosarcoma | II | yes | positive | TP53 | yes | 2 | 2/0 | 1,3 |
| 15 | Alveolar rhabdomyosarcoma | IIIb | yes | NA | NA | No cfDNA target | NA | NA | NA |
| 16 | Desmoplastic small round cell tumor (DSRT) | IV | yes | NA | NA | No cfDNA target | NA | NA | NA |
| 17 | Alveolar rhabdomyosarcoma | IIIB | yes | NA | NA | No cfDNA target | NA | NA | NA |
| 18 | Synovial sarcoma | IV | yes | NA | NA | No cfDNA target | NA | NA | NA |
| 19 | Epitheloid sarcoma | III | no | NA | NA | No cfDNA target | NA | NA | NA |
| 20 | Leiomyosarcoma | IV | yes | NA | NA | No cfDNA target | NA | NA | NA |

Footnote: The table includes all 20 screened patients; patients 1–14 underwent longitudinal ctDNA monitoring, whereas patients 15–20 had no suitable target for panel-based monitoring. Disease stage refers to monitoring initiation. ctDNA detection indicates detection at any time during monitoring. Concordant/discordant assessments are reported as the respective numbers of paired ctDNA–imaging assessments with matching/differing PD versus non-PD classifications. Treatment codes: 1, systemic antitumor therapy; 2, radiotherapy; 3, surgery. Missing baseline samples and lack of a suitable monitoring target must not be interpreted as negative ctDNA results. ctDNA, circulating tumor DNA; PD, progressive disease; UICC, Union for International Cancer Control; NA, not applicable or not available, as appropriate.

#### **Supplementary Table S3: Timing of blood collection in relation to radiological assessment**

| Patient | Number of paired ctDNA–imaging assessments | Individual sampling–imaging intervals (days) | Range of interval between paired measurements (days) | Median of interval between paired measurements (days) |
| --- | --- | --- | --- | --- |
| 1 | 10 | +2,0, -7, 0, 0, -10, -14. +34, -3, -20 | −20 to +34 | -1,5 |
| 2 | 5 | +6, +8, 0, -20, +8 | −20 to +8 | +6 |
| 3 | 4 | 0, -15, +22, +7 | −15 to +22 | +3.5 |
| 4 | 3 | +14, -10, +8 | −10 to +14 | +8 |
| 5 | 5 | 0, 0, -8, 0, -18 | −18 to 0 | 0 |
| 6 | 4 | 0, +12, 0, +16 | 0 to +16 | +6 |
| 7 | 4 | +5, -20, -17, +9 | −20 to +9 | -6 |
| 8 | 2 | -7, +10 | -7 to +10 | +1,5 |
| 9 | 5 | +7, +3, +8, +18, 0 | 0 to +18 | +7 |
| 10 | 7 | +21, +4, -9, +29, +10, +2, +6 | −9 to +29 | +6 |
| 11 | 9 | +18, +31, +14, +11, +10, -16, -5, +12, +4 | −16 to +31 | +11 |
| 12 | 3 | 0, -1, -2 | −2 to 0 | −1 |
| 13 | 4 | 1, -4, +10, -9 | −9 to +10 | −2.5 |
| 14 | 2 | +4, +23 | +4 to +23 | +13.5 |

Footnote: Intervals were calculated as the date of blood collection minus the date of the paired radiological examination (day 0). Negative values indicate blood collection before imaging, positive values indicate blood collection after imaging, and zero indicates same-day assessments. Individual intervals are listed chronologically for each patient. The median and range (minimum–maximum) were calculated from the signed sampling–imaging intervals, retaining negative and positive values. ctDNA, circulating tumor DNA.

#### **Supplementary Table S4: Discordant assessments**

| Sample | Histology | Disease stage (UICC) | Disease Site | Timing between sampling and imaging (days) | Treatment exposure | ctDNA finding at paired assessment | Subsequent imaging | Possible explanation for discordance |
| --- | --- | --- | --- | --- | --- | --- | --- | --- |
| 1 | Ewing sarcoma | IV | multifocal | 0 | 1,3 | negative | PD with new brain metastases | new brain metastases after the primary tumor resection |
| 2 | Ewing sarcoma | IV | multifocal | +34 | 1 | negative | PD | large time gap between samples |
| 3 | Ewing sarcoma | IIb | Thoracic wall | +10 | none | negaitve | SD | Unclear |
| 4 | Osseus Ewing sarcoma | IV | multifocal | +12 | 1 | negative | PD | Suspect pleural tumor progression, subsequent positive ctDNA and pulmonary progession |
| 5 | Osseus Ewing sarcoma | IV | multifocal | +10 | 1 | TP53 (c.535) | SD | Radiological progresson at the subsequent imiging |
| 6 | Desmoplastic small round cell tumor (DSRT) | IV | multifocal | +10 | 1 | negative | PD | Unclear ; ctDNA levels below the assay detection limit are a possible explanation |
| 7 | Desmoplastic small round cell tumor (DSRT) | IV | multifocal | +2 | 1 | negative | PD | Unclear, ctDNA levels below the assay detection limit are a possible explanation |
| 8 | Desmoplastic small round cell tumor (DSRT) | IV | multifocal | +6 | 1 | negative | PD | Unclear, ctDNA levels below the assay detection limit are a possible explanation |

Footnote: Each row represents one discordant paired assessment, defined as differing ctDNA-based and radiological PD/non-PD classifications. Individual patients may contribute more than one discordant assessment. Sampling–imaging intervals were calculated as the blood collection date minus the imaging acquisition date; negative values indicate sampling before imaging, positive values indicate sampling after imaging, and zero indicates same-day assessments. Treatment codes: 1, systemic antitumor therapy; 2, radiotherapy; 3, surgery. “Negative” indicates that ctDNA was not detected above the assay-specific calling threshold. Subsequent imaging refers to follow-up imaging after the paired radiological examination. Possible explanations for discordance are descriptive interpretations; “unclear” indicates that the available data did not establish an explanation. ctDNA, circulating tumor DNA; PD, progressive disease; SD, stable disease; UICC, Union for International Cancer Control.

#### **Supplementary Table S5: CancerDetect UMI Panel (CFD v.2) targets**

| Gene | Transcript (NM_#) | Exons/Region targeted | Example hotspot mutations in target |
| --- | --- | --- | --- |
| AKT1 | NM_005163.2 | Exon 2 | E17 |
| ALK | NM_004304.5 | Exons 22-25 | F1174, G1202, F1245, R1275 |
| AR | NM_000044.6 | Exons 4, 5, 8 | - |
| BRAF | NM_004333.6 | Exons 11 and 15 | V600 |
| CDKN2A | NM_000077.5 | entire coding region | - |
| CTNNB1 | NM_001904.4 | Exons 2, 6, 7 | S37, S45, K335, N387 |
| EGFR | NM_005228.5 | Exons 2, 3, 6, 7, 15, 18-21 | A289, G598, E746_A750del, T790, L858 |
| ERBB2 | NM_004448.4 | Exons 8, 17, 19-21 | S310, R678, V842 |
| ERBB3 | NM_001982.4 | Exons 3, 7-9, 23 | V104, E928 |
| ESR1 | NM_000125.4 | Exons 4, 5, 7, 8 | K303, Y537, D538, E380Q, L536H, Y537C/N/S, D538G |
| FGFR1 | NM_023110.3 | Exons 11-13 | N577, K687 |
| FGFR2 | NM_000141.5 | Exons 6, 8, 11-13 | S252, N549 |
| FGFR3 | NM_000142.5 | Exons 6, 8, 13 | R248, S249, Y375 |
| GNA11 | NM_002067.5 | Exons 4, 5 | R183, Q209 |
| GNAQ | NM_002072.5 | Exons 2, 4, 5 | T96, R183, Q209 |
| GNAS | NM_000516.7 | Exon 8 | R201 |
| H3-3A | NM_002107.7 | Exon 1 | K27, G34 |
| HRAS | NM_005343.4 | Exons 1-3 | G12, Q61 |
| IDH1 | NM_005896.4 | Exon 2 | R132 |
| IDH2 | NM_002168.4 | Exon 4 | R140, R172 |
| JAK2 | NM_004972.4 | Exon 12 | V617 |
| KIT | NM_000222.3 | Exons 9, 11, 13, 14, 17 | W557_K558del, D816 |
| KRAS | NM_004985.5 | Exons 1-3 | G12, G13, Q61 |
| MET | NM_001127500.3 | Exons 13, 15, 18 | L982_D1028del, T1010, Y1248, Y1253, MET-Exon-14-skipping |
| NRAS | NM_002524.5 | Exons 1-3 | G12, Q61 |
| PDGFRA | NM_006206.6 | Exons 11, 13, 17 | D842 |
| PIK3CA | NM_006218.4 | Exons 1, 4, 7, 9, 13, 20 | R93, HS E542, E545, H1047 |
| PTEN | NM_000314.8 | entire coding region | - |
| RET | NM_020975.6 | Exons 10, 11, 13-16 | C634 |
| TERT | NM_198253 | Promotor | c.-124 (C228), c.-146 (C250) |
| TP53 | NM_000546.6 | entire coding region | - |

Footnote: Exon numbering refers to the coding exons of the indicated reference transcript. Listed hotspot positions and variants are illustrative examples within the targeted regions and do not constitute an exhaustive list or indicate the alterations monitored in individual patients. A dash indicates that no specific hotspot example is listed, rather than absence of target coverage. UMI, unique molecular identifier; NM_, RefSeq messenger RNA accession prefix.

#### **Supplementary Figure S1: Descriptive Kaplan–Meier estimate of progression-free survival from initial sarcoma diagnosis**

#### Kaplan–Meier estimate of progression-free survival (PFS) in the screened cohort of 20 patients, measured from initial sarcoma diagnosis to the first documented radiological or clinical progression or death from any cause, whichever occurred first. Patients without an event were censored at the date of last follow-up; tick marks indicate censored observations. Time is shown in months. This descriptive analysis characterizes the disease course of the study cohort and was not designed to assess the prognostic value of ctDNA monitoring or treatment-line-specific outcomes. ctDNA, circulating tumor DNA.

#### **Supplementary Figure S2: Descriptive Kaplan–Meier estimate of overall survival from initial sarcoma diagnosis**

Kaplan–Meier estimate of overall survival (OS) in the screened cohort of 20 patients, measured from initial sarcoma diagnosis to death from any cause. Patients without a documented death by the data cutoff were censored at the date of last follow-up; tick marks indicate censored observations. Time is shown in months. This descriptive analysis characterizes survival in the study cohort and was not designed to assess the prognostic value of ctDNA monitoring. ctDNA, circulating tumor DNA.
